# A Bibliometric Review of Publications on Innovative Behaviors of Nurse Managers

**DOI:** 10.1155/jonm/5950542

**Published:** 2025-04-21

**Authors:** Nurcan Bi̇lgi̇n, Damla Şahi̇n Büyük, Sevgi Paki̇ş Çeti̇n

**Affiliations:** ^1^Department of Nursing Management, Manisa Celal Bayar University, Manisa, Turkey; ^2^Department of Public Health Nursing, Manisa Celal Bayar University, Manisa, Turkey; ^3^Department of Fundamentals of Nursing, Manisa Celal Bayar University, Manisa, Turkey

**Keywords:** bibliometric analysis, innovative behavior, nurse managers, nursing, VOSviewer

## Abstract

**Aim:** This study used bibliometric indicators to provide an overview of research on nurse managers' innovative behaviors.

**Background:** Encouraging nurses' innovative abilities leads to improved care quality and work efficiency and reduced healthcare costs. For these reasons, the innovative behaviors of nurse managers are crucial for both their personal development and the success of their teams.

**Method(s):** Bibliometric methods were employed to investigate the innovative behaviors of nurse managers. Data were collected from the Web of Science (WoS) database through mid-September 2024. A query string search yielded 478 articles, of which 44 met the inclusion criteria and were analyzed using bibliometric techniques. Microsoft Excel and the VOSviewer software were used to analyze the distribution of publications by year, leading countries, prominent journals, contributing authors, coauthor networks, and keyword co-occurrence.

**Results:** There has been a recent increase in publications on the innovative behaviors of nurse managers, with the United States leading in publication output. The Journal of Nursing Management and the Journal of Nursing Administration were identified as the most prolific journals in this area. In the coauthorship analysis, nine authors emerged as the most linked, forming a cluster with a total of 36 links. Furthermore, the most used keywords were “nurses,” “psychological empowerment,” and “innovative behavior.” Additionally, 56.8% of the articles employed quantitative methods, 31.8% used qualitative methods, and 11.4% applied mixed-method research designs.

**Conclusion(s):** The analysis of articles indexed in the WoS database suggests that research on the innovative behaviors of nurse managers is still in its developmental stages.

**Implications for Nursing Managers:** Future research should explore the innovative behaviors of nurse managers and the factors influencing these behaviors further. Both qualitative and quantitative studies should examine how and to what extent these behaviors impact the nurses under their supervision.

## 1. Introduction

In today's healthcare landscape, organizations must embrace innovation to achieve their objectives. Innovation is crucial for healthcare organizations to respond to, challenge, and adapt to changes to survive and remain competitive [1]. Within this context, the actions of employees play a critical role in fostering continuous innovation and improvement. While employee creativity involves generating new and useful ideas related to products, services, processes, and procedures, innovative work behavior includes the implementation of these ideas as well [2]. Nurses, positioned at the forefront of care delivery, are critical thinkers who frequently innovate by identifying more efficient processes or repurposing items for alternative uses. Given their unique role in advancing clinical practice, nurses must continually engage in innovation to enhance the quality of healthcare delivery [3, 4]. The American Nurses Association (ANA) defines nursing innovation as the ability to proactively seek and develop new methods, technologies, and tools to promote health, prevent disease, improve patient care quality, and foster teamwork through rational support channels [5]. Several individual-level factors, such as sociodemographic characteristics such as the education level, job position, professional title, salary, and nursing experience, are critical in shaping nurses' innovative behaviors [6, 7]. Nurse managers, who lead hospital departments or units, play a vital role in promoting innovation within their teams. Their innovative behaviors not only enhance their leadership, work efficiency, and nursing quality but also improve the overall competitiveness of the nursing team, encouraging innovative thinking and behaviors among staff [8]. Encouraging the innovative capabilities of nurses can lead to significant improvements in healthcare outcomes, including the enhanced quality of medical services and treatment effectiveness, increased work efficiency, reduced healthcare costs while meeting patient needs, improved care service effectiveness, better access to health services, and the simplification of healthcare delivery processes [4, 9, 10]. For example, in a public hospital in Turkey, the innovation process in nursing was initiated in 2012 under the leadership of the head nurse and was strengthened through various training sessions and awareness meetings. Additionally, as part of the hospital's 150th anniversary celebrations, a project competition and symposium titled “Innovation in Nursing” were organized to encourage innovation in nursing. This innovation process continued traditionally for 7 years within the hospital, and during this period, the contributions of the nurses were crucial. With the philosophy of “The Best Person Knows the Job,” a project development team was formed from nurses providing 24/7 service in the field. This team, during their innovation journey, developed 376 medical inventions, particularly aimed at supporting maternal and infant health. Over the 7-year period, patent and utility model applications were made for the innovative inventions produced by the nurses, and about 50 patent certificates were obtained [11]. This paragraph provides a comprehensive example of how the head nurse's adoption of an innovative work behavior and their encouragement of staff to think and act innovatively led to the initiation and continuation of the innovation process in nursing. This example serves as an important model for the professional development of nurses and the improvement of healthcare services. Involving nurses in innovative processes not only enhances their professional satisfaction but also contributes to the healthcare system. For these reasons, it is crucial to investigate the innovative behaviors of nurse managers and clearly demonstrate their impact on healthcare services. In this context, the bibliometric analysis method serves as a powerful tool for exploring trends and assessing the scientific productivity of research [12]. Bibliometric analysis, which is the structured approach to comprehending the dynamics of a particular field, has advantages in predicting the forward-looking trends of disciplines [13]. A bibliometric analysis of studies on nurse managers' innovative behaviors aims to make the existing body of knowledge more visible while providing key insights that can guide future research. This study aims to analyze research on the innovative behaviors of nurse managers using bibliometric indicators, thereby summarizing studies and enhancing the visibility of their findings.

## 2. Methods

### 2.1. Design

In this study, bibliometric methods were employed to evaluate research on the innovative behaviors of nurse managers. Bibliometric studies provide a robust foundation for advancing a field in meaningful ways by enabling researchers to gain an overview of the literature, identify knowledge gaps, and generate new research ideas [14]. Bibliometric methodology involves the application of quantitative techniques (such as citation analysis) to bibliometric data [15]. Today, bibliometric analysis is recognized as a global approach for assessing the current state of research across various disciplines [16]. The bibliometric method was used in this study to provide an in-depth understanding of research on the innovative behaviors of nurse managers.

### 2.2. Search Strategy and Data Collection

Data for this bibliometric analysis were collected from the Web of Science (WoS) database between July and mid-September in 2024. Using the advanced search tool in the WoS database, specific terms were entered into the ‘topic' field, and filters were applied to refine the results.

The primary search keywords included variations of ‘innovative' combined with terms such as “innovat^∗^ or innovative behavior or innovative behaviour or innovative work behavior, or innovative work behaviour or innovation behavior, or innovation behaviour” and “nurs^∗^ manager^∗^ or nurs^∗^ supervisor or nurs^∗^ administ^∗^ or charge nurs^∗^ or nurs^∗^ leader^∗^ or head nurs^∗^ or nurs^∗^ exec^∗^ or chief nurs^∗^ or direct nurs^∗^.” The study focused on research articles published between 1992 and mid-September 2024.

**Figure 1:**
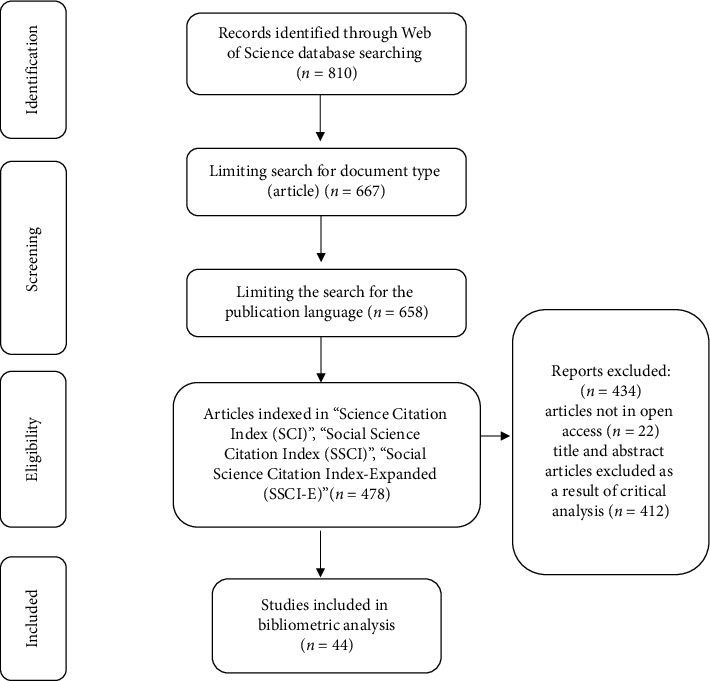
PRISMA flow diagram of the study selection process.

The initial query string search identified 810 articles. The scope of this study was confined to full-text, open-access articles published in English-language journals indexed in the SCI, SCIE, and SSCI databases. Excluded from this bibliometric analysis were books, book chapters, conference proceedings, reviews, letters to the editor, review articles (*n* = 143), and non-English language publications (*n* = 9). Subsequently, 478 articles published in English-language journals indexed in SCI, SCIE, and SSCI indexes were accessed. After excluding non–open-access articles (*n* = 22), titles and abstracts of these articles were thoroughly reviewed, with full texts examined as necessary. The evaluation of the retrieved articles was conducted independently by two researchers in a blinded process, and any discrepancies were resolved through discussion. Of the 456 articles reviewed, 412 were excluded due to irrelevance to the research topic. Ultimately, 44 articles considered directly relevant were included in the bibliometric analysis (Figure 1).

### 2.3. Data Analysis

VOSviewer is a powerful tool designed specifically for creating and displaying bibliometric maps. It excels in visualizing large datasets in a way that is both accessible and interpretable, making it ideal for bibliometric analysis. Moreover, VOSviewer can be used free of charge [17]. On the other hand, Microsoft Excel is the widely used spreadsheet software that offers extensive data analysis and documentation capabilities. It organizes data in a grid of rows and columns, with each intersection referred to as a “cell.” Excel is especially valuable in bibliometric analysis, as it efficiently handles file formats such as “.csv,” which are commonly exported from databases such as WoS [18]. Bibliometric and visualization analyses were conducted using both Microsoft Excel and the VOSviewer software. The distribution of publications by country and year was analyzed using Excel, with corresponding graphs and figures generated to present the findings. VOSviewer was employed to perform coauthorship analysis, source-citation analysis to identify leading journals, and co-occurrence analysis of author keywords.

## 3. Results

**Figure 2:**
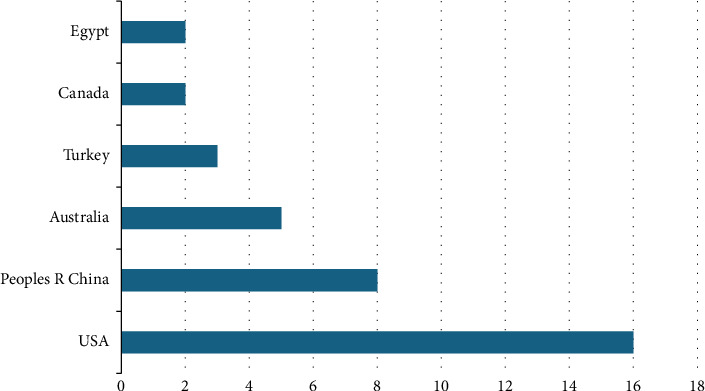
Distribution of publications by countries.

The distribution of countries with the highest number of publications on innovative behaviors of nurse managers is presented in Figure 2. The United States had the highest publication output with 16 articles, followed by China (8), Australia (5), Turkey (3), Canada (2), and Egypt (2), all of which made significant contributions. Additional countries contributing to this field include Brazil, England, France, Germany, Iran, Italy, Jordan, Kenya, Mauritius, Northern Ireland, Pakistan, Saudi Arabia, South Korea, Spain, and Thailand with one publication.

**Figure 3:**
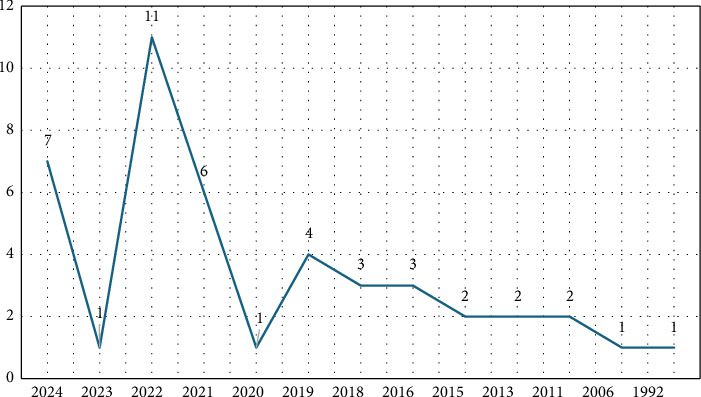
Distribution of publications by years.

Figure 3 illustrates the annual distribution of publications on the innovative behaviors of nurse managers from the WoS database. The first article on this topic was published in 1992. A period of stagnation in the literature occurred between 2006 and 2011. However, a surge in publications began after 2011, with a significant increase starting in 2016. By 2022, the number of publications peaked, with the exception of 2020 and 2023, when there was a decline.

**Figure 4:**
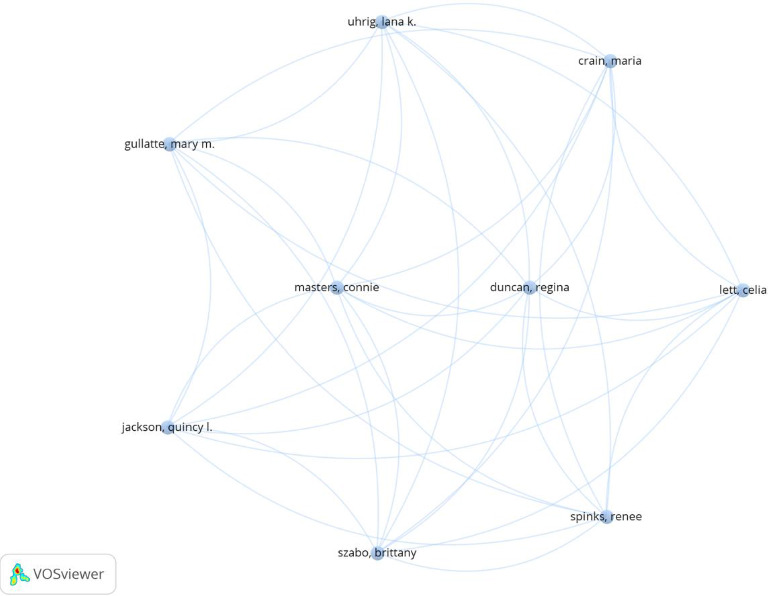
Graphical representation of coauthor analysis.

The coauthorship analysis of authors is visualized in Figure 4, where a network map was generated to identify the most interconnected authors. The analysis applied the criteria of at least one publication and at least one citation. Out of this analysis, nine authors emerged as the most connected, forming a cluster with 36 total connections.

**Figure 5:**
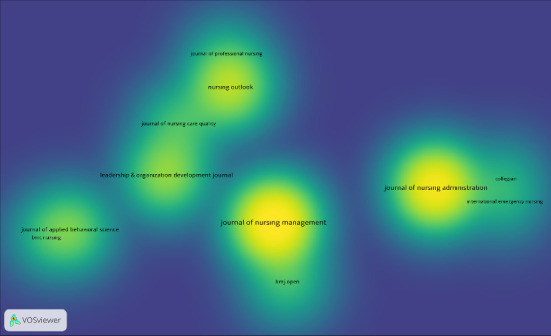
Distribution of publications by journals.

In terms of journal distribution, articles on the innovative behaviors of nurse managers were published in 26 scientific journals. Of these, 43.2% appeared in nursing-specific journals, while 29.5% were published in the *Journal of Nursing Management*, which spans nursing and management categories in the WoS. The journals with the highest number of publications on this topic are shown in Figure 5. While the first original research on the innovative behaviors of nurse managers was published in Advances in Nursing Science, the Journal of Nursing Management, which has a 5-year impact factor of 4.5 and classified in the Q1 impact factor category, led in terms of publication output on this subject, with 13 articles. Similarly, the Journal of Nursing Administration, with a 5-year impact factor of 2 and in the Q2 category, followed with 6 articles.

**Figure 6:**
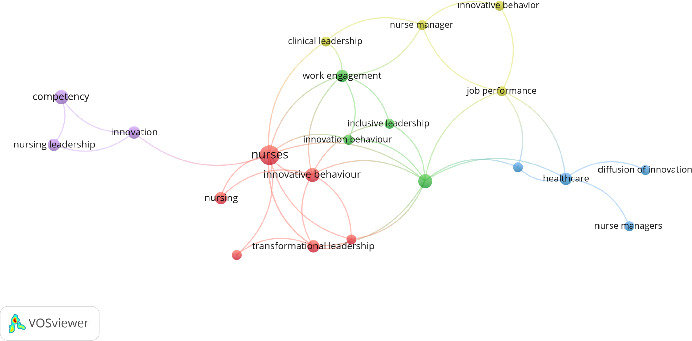
Graphical representation of authors' keyword co-occurrence in the overlay visualization mode.

Figure 6 presents the results of a term analysis as a form of a word cloud, displaying the keywords used in the analyzed publications. A total of 138 distinct keywords were identified. When a threshold of “used at least two times” was set, 21 frequently occurring keywords were identified. The analysis of the map formed with these keywords revealed five major clusters and 41 connections. The most used keywords were “nurses,” “psychological empowerment,” and “innovative behavior.”

Table 1 lists the five most-cited articles related to the innovative behaviors of nurse managers. The most cited studies were by Birken et al. (2016), Afsar and Masood (2018), Wang et al. (2019), Xerri (2013), and White et al. (2016), respectively. Furthermore, the research designs employed in the articles were categorized, with 56.8% being quantitative, 31.8% qualitative, and 11.4% mixed-method studies. Quantitative studies were published between 2011 and 2024, qualitative studies between 1992 and 2024, and mixed-method studies between 2013 and 2021.

## 4. Discussion

The innovative behaviors of nurse managers are not only pertinent to their actions but are also closely tied to their leadership role within the team. Nurse managers who think and act innovatively serve as role models, encouraging their team members to adopt similar behaviors. Fostering innovation among nurses has been linked to several positive outcomes, such as improving the quality of care and enhancing work efficiency. Therefore, it is crucial to investigate the innovative behaviors of nurse managers. This bibliometric study aimed to assess current research on the topic using data from the WoS database. Out of an initial 810 articles, 44 that met the inclusion criteria were selected for detailed analysis.

The findings reveal that most research in this area has been conducted in the United States, China, and Australia. Collaborative publications were also noted, with Australia partnering with the United Kingdom, the United States, and Iran; Canada with Brazil and Kenya; Egypt with Saudi Arabia; and France with Italy. However, many countries show limited or no interest in this area of research. This lack of focus on the innovative behaviors of nurse managers as role models is concerning, particularly given the critical need for innovation within healthcare institutions to remain competitive and effective.

An examination of the publication timeline reveals that research on the innovative behaviors of nurse managers has been sporadic, and the relevant articles were published between 1992 and 2024. From 1992 to 2006, no original research on this subject was found, and between 2006 and 2011, no new studies were published in the WoS database. However, after 2011, there was a significant increase in research activity, culminating in the highest number of publications in 2022. By mid-September 2024, seven publications had been recorded, suggesting that the total number for 2024 may closely match that of 2022. These findings indicate that research on this topic remains limited in scope and is still in a developmental phase.

Coauthorship analysis plays a crucial role in bibliometric studies as it offers valuable insights into the dynamics of collaboration among authors, universities, and countries. It reveals the structure and dynamics of these networks by identifying who collaborates on research projects and publications [19]. Furthermore, coauthorship analysis, which is recognized as an indicator of collaboration in scientific publications, indicates a strong social bond [20]. In this study, the coauthorship analysis revealed a network of 9 authors connected through 36 links, forming a single cluster. Notably, except for one author, all the authors within this cluster were affiliated with the same institution and country. Interestingly, the five most highly cited authors, as listed in Table 1, did not appear among the most connected authors. This suggests that these highly cited researchers either work more independently or that their work exerts a broad impact without being integrated into a specific collaborative network. While individual citations are crucial for measuring scientific impact, collaboration also plays a critical role in enhancing scholarly influence. Therefore, fostering new partnerships between researchers and expanding collaborations across institutions and countries can significantly broaden and strengthen scientific networks.

Citation analysis, a measurement tool that counts how many times a publication has been cited by other publications and determines its significance, helps predict the impact of documents, authors, or journals and quickly identifies important studies in a field [20, 21]. Research on the innovative behaviors of nurse managers sits at the intersection of multiple disciplines, including nursing, health management, leadership, and organizational behavior, offering a broad perspective that contributes to scholarly discussions across these fields [22]. This study found that articles on this topic were published in 26 scientific journals, with 17 focused on nursing, five in social sciences, and four in health-related fields. The prevalence of publications in journals categorized under nursing and management highlights the interdisciplinary nature of this subject. Moreover, exploring research on this topic within nursing is likely to contribute significantly to the profession. Nearly half of the articles on the innovative behaviors of nurse managers were published in the Journal of Nursing Management and the Journal of Nursing Administration. The prominence of these journals in publishing research on this topic reflects the critical role that nurse managers play in fostering innovation within both nursing and health management. These journals serve as pioneers in raising awareness about the need for nursing leaders to develop innovative solutions, which is vital for advancing healthcare practices.

The examination of the co-occurrence of keywords necessary to define the content of documents is called coword analysis. This analysis is used to determine the degree to which keywords and concepts related to the research area occur together [23]. In the keyword analysis, the most frequently used terms were nurses, psychological empowerment, and innovative behavior. In the literature, the concept of ‘nurse manager' is expressed with various terms (e.g., nurse executives, chief nurse, nurse manager, and head nurse). Due to this diversity, it is believed that the concept of ‘nurse manager' has been mentioned less frequently in the keyword analysis compared to ‘nurse'. However, this term diversity actually reflects the richness and variety of the literature in this field. Additionally, the frequency of terms such as ‘innovative behavior' and ‘psychological empowerment' in the keyword analysis indicates a strong research trend in these areas. Interestingly, the higher frequency of the term ‘psychological empowerment' suggests that innovative behavior has been researched more frequently in relation to the psychological empowerment variable. Moreover, the innovative behaviors of nurse managers were often examined in conjunction with concepts such as leadership, job commitment, and job performance, rather than exclusively focusing on innovation and nursing. Sritoomma and Wongkhomthong (2021) conducted a qualitative study aimed at identifying the components of strategic leadership competencies in head nurse managers. They identified the theme of “Strategic-Innovation Thinking and Planning” [24]. Haddad et al. conducted a study to explore the perceptions of nurse unit managers regarding their work and leadership practices within a large, multifacility tertiary healthcare organization. In this study, the clinical leadership profile was evaluated in five main categories, one of which included the category of “Drives Innovation” [25]. These studies suggest that the innovative behaviors of nurse managers are closely related to their leadership roles. The prominence of subdimensions such as “innovative thinking and planning” and “innovation” in articles investigating the leadership competence of nurse managers likely contributed to the emphasis on leadership in the keyword analysis.

In this bibliometric study, an evaluation of the five most-cited articles revealed that three were published in social science journals indexed in the WoS, while two appeared in nursing journals. Notably, the journal with the highest number of publications on this topic ranked third in terms of citation frequency. The *Journal of Nursing Management* published two highly cited articles in 2011, and although these appeared before 2019, they remain among the top 10 most-cited articles in the field. In terms of research methodologies, a trend was observed in qualitative designs in earlier studies examining the innovative behaviors of nurse managers (research conducted between 1992 and 2011). Qualitative research is primarily concerned with understanding meaning, exploring the content of practices, identifying unexpected phenomena, analyzing processes, and developing causal explanations. It is often employed when there is limited research on a subject and is used as a precursor to quantitative studies [26]. Review articles on the innovative behaviors of nurse managers were identified as early as 1992 and 1993. Literature reviews are often described as a systematic method of gathering and synthesizing prior research, providing a robust foundation for advancing knowledge and supporting theory development [27]. The primary goal of such reviews is to either summarize specific topics or offer a comprehensive overview of broader areas of inquiry [28]. In this context, it is expected that the innovative behaviors of nurse managers were initially explored through qualitative studies and reviews, serving as preparation for subsequent quantitative research.

### 4.1. Limitations

The most significant limitation of this study is that the analyses were restricted to studies listed in the WoS database, excluding sources not available online through other databases such as TUBITAK Ulakbim, the YÖK Thesis Archive in Turkey, and Scopus and PubMed internationally. Additionally, the study was limited to English-language and open-access research articles.

## 5. Conclusion

Professional characteristics such as the position, job title, and nursing experience influence nurses' innovative behaviors at an individual level, while the innovative behaviors of nurses in managerial roles are closely linked to the teams they lead. Investigating the innovative behaviors of nurse managers, who serve as role models in various areas, is therefore crucial.

With the increasing role of technology in healthcare, the need for innovation has grown, and recent years have seen more publications on this topic. However, an analysis of articles indexed in the WoS revealed a limited number of studies on this subject. It appears that both countries and researchers have shown limited interest in the innovative behaviors of nurse managers. As a result, the research in this area is still in its early stages and lacks sufficient maturity.

### 5.1. Implications for Nurse Managers

Based on the findings, it is recommended to further investigate the intrinsic and extrinsic motivational sources of nurses to understand what drives their innovative behaviors. This will help develop strategies to better encourage nurses' innovative behaviors. Additionally, to foster innovative behaviors, it is suggested that nurse managers receive continuous education and develop their leadership skills. In these years when digitalization in healthcare is at the forefront, it is of great importance to research the contributions of innovative-thinking nurse managers to both institutions and society. Furthermore, it is recommended to conduct qualitative and quantitative studies on how and to what extent nurse managers' innovative behaviors impact the nurses they are responsible for.

## Figures and Tables

**Table 1:** Top five quotes in the field of nurse managers' innovative behavior.

Year	Author name	Title	Journal	Citation
2016	Birken, S. A., DiMartino, L. D., Kirk, M. A., Lee, S.Y. D., McClelland, M., Albert, N. M.	Elaborating on the theory with middle managers' experience implementing healthcare innovations in practice	Implementation Science	241
2018	Afsar, B., Masood, M.	Transformational leadership, creative self-efficacy, trust in the supervisor, uncertainty avoidance, and innovative work behavior of nurses	Journal of Applied Behavioral Science	108
2019	Wang, Y. X., Yang, Y. J., Wang, Y., Su, D., Li, S. W., Zhang, T., Li, H. P.	The mediating role of inclusive leadership: work engagement and innovative behavior among Chinese head nurses	Journal of Nursing Management	64
2013	Xerri, M.	Workplace relationships and the innovative behavior of nursing employees: a social exchange perspective	Asia Pacific Journal of Human Resources	56
2016	White, K. R., Pillay, R., Huang, X.	Nurse leaders and the innovation competence gap	Nursing Outlook	31

## Data Availability

The data that support the findings of this study are available from the corresponding author upon reasonable request.
